# Elevated Temperature Alters the Lunar Timing of Planulation in the Brooding Coral *Pocillopora damicornis*

**DOI:** 10.1371/journal.pone.0107906

**Published:** 2014-10-15

**Authors:** Camerron M. Crowder, Wei-Lo Liang, Virginia M. Weis, Tung-Yung Fan

**Affiliations:** 1 Department of Integrative Biology, Oregon State University, Corvallis, Oregon, United States of America; 2 Institute of Marine Biology, National Dong Hwa University, Pingtung, Taiwan, R.O.C.; 3 National Museum of Marine Biology and Aquarium, Pingtung, Taiwan, R.O.C.; Pennsylvania State University, United States of America

## Abstract

Reproductive timing in corals is associated with environmental variables including temperature, lunar periodicity, and seasonality. Although it is clear that these variables are interrelated, it remains unknown if one variable in particular acts as the proximate signaler for gamete and or larval release. Furthermore, in an era of global warming, the degree to which increases in ocean temperatures will disrupt normal reproductive patterns in corals remains unknown. *Pocillopora damicornis,* a brooding coral widely distributed in the Indo-Pacific, has been the subject of multiple reproductive ecology studies that show correlations between temperature, lunar periodicity, and reproductive timing. However, to date, no study has empirically measured changes in reproductive timing associated with increased seawater temperature. In this study, the effect of increased seawater temperature on the timing of planula release was examined during the lunar cycles of March and June 2012. Twelve brooding corals were removed from Hobihu reef in Nanwan Bay, southern Taiwan and placed in 23 and 28°C controlled temperature treatment tanks. For both seasons, the timing of planulation was found to be plastic, with the high temperature treatment resulting in significantly earlier peaks of planula release compared to the low temperature treatment. This suggests that temperature alone can influence the timing of larval release in *Pocillopora damicornis* in Nanwan Bay. Therefore, it is expected that continued increases in ocean temperature will result in earlier timing of reproductive events in corals, which may lead to either variations in reproductive success or phenotypic acclimatization.

## Introduction

Reproductive timing is a critical factor in coral reproductive success and has been correlated to multiple environmental variables including those associated with seasonality such as temperature, solar irradiance, tidal cycles, nocturnal illumination associated with lunar periodicity, and light-dark cycles corresponding with diel fluctuations [1]–[8]. Previous studies have effectively demonstrated correlations between environmental variables and coral reproduction; however, examining the direct causality of individual variables on the timing of reproduction is critical to understanding the mechanisms controlling reproductive timing. Determining which variables are vital for the coordination of reproductive events will reveal information underlying coral reproductive function. Corals are both the bio-engineers and foundational primary producers of coral reef ecosystems and therefore, understanding how environmental variables affect timing of reproduction is essential for predicting future impacts of climate change on coral reef ecosystem stability.

Temperature has been shown to be a critical variable affecting coral reproductive success. Corals of all life stages are negatively affected by increasing sea-surface temperature attributed to global climate change [9]–[11]. While significant effort has been dedicated to describing the effects of temperature on the physiology and ecology of corals, less attention has focused on the effects of elevated temperature on coral reproductive timing and success [9], [12]. Recent studies have revealed that temperatures exceeding tolerance thresholds reduce polyp fecundity, gametic quality [13], [14] and the number of reproductive events in corals [15]. While it is evident that elevated temperatures can impair reproductive processes, predictable seasonal fluctuations in temperature might be a key component controlling the timing of reproduction. Multiple field studies have observed correlations between temperature and reproductive timing [16], [17]. This correlation could demonstrate that some corals are displaying reproductive plasticity or alterations in the timing of release to adapt to change, which could be an important mechanism for larval survival and fitness with rising ocean temperatures associated with climate change. Understanding how temperature affects the timing of reproduction will provide information as to how continued increases in sea-surface temperature may further alter reproductive processes and patterns in corals.

Approximately 15% of coral species brood internally fertilized larvae (planulae) that are released during a process known as planulation. Planulation typically occurs multiple times annually and in some cases on a monthly basis [18], [19]. Planulae are often buoyant and have the capacity for wide dispersal, but are also able to quickly settle upon release leading to fast rates of colonization [2], [20]. The timing of planulation has a direct influence on larval survival, dispersal, and recruitment [2], [19]. Reproductive events optimally timed to environmental variables such as temperature and light, can affect larval survival particularly in larvae containing symbiotic dinoflagellates [21]–[24].

The widely studied brooding coral *Pocillopora damicornis*, has been the subject of multiple studies investigating reproduction, particularly reproductive synchrony associated with lunar periodicity [19], [20], [25]–[27]. Collectively, these observations show variability in planulation patterns. *P. damicornis* has been shown to release planulae at every lunar phase during monthly lunar reproductive cycles with some consistencies observed within similar geographical locations. This diversity in the timing of planulation between different geographical regions may be the result of phenotypic plasticity that increases fitness [28]. However, to date, little is known about phenotypic plasticity of coral reproductive timing, and specifically how this plasticity may be driven by environmental variables, such as rising ocean temperatures.

In the context of the ongoing debate as to the effect of individual environmental variables on the timing of planulation, it is timely to ask if changes in temperature can directly alter planulation patterns in *P. damicornis*. The purpose of this study was to determine the effect of increased seawater temperature on the timing of planulation within a single lunar cycle and between lunar cycles of different seasons. To investigate this, *P. damicornis* colonies were monitored in temperature-controlled tanks exposed to natural lunar cycles with seawater temperature set to either 23°C (low) or 28°C (high). For both seasons, the timing of planulation was found to be plastic, with elevated temperature treatments resulting in significantly earlier peaks of planula release. This suggests that temperature modifies the lunar timing of larval release in *Pocillopora damicornis* in Nanwan Bay.

## Materials and Methods

### Coral Collection

Twelve adult colonies of *P. damicornis* were randomly collected with permission from the Kenting National Park, Taiwan from a 10-meter area at Hobihu reef (21°56.799′N, 120°44.968′E) in Nanwan Bay, Taiwan on two separate occasions in lunar March and June 2012. Colonies ranged in size from 7–15 cm in diameter and were removed from reefs 4–5 meters in depth. Within an hour after collection, colonies were transported back to The National Museum of Marine Biology and Aquarium (NMMBA) in Checheng, Taiwan.

### Experimental Design

Six 150 liter tank mesocoms situated within a larger (30 ton) aquarium system as described by Mayfield et al. (2013) were used in this study. Three of the tanks were maintained at 23°C (mean winter ocean temperature) and three were maintained at 28°C (mean summer ocean temperature). Two coral colonies were randomly selected and assigned to each tank. Tanks were covered with shade cloth and exposed to natural outdoor sunlight and received a constant supply of sand-filtered seawater. Corals were allowed to acclimate to the experimental temperature conditions for one week prior to monitoring of planulation.

**Figure 1:**
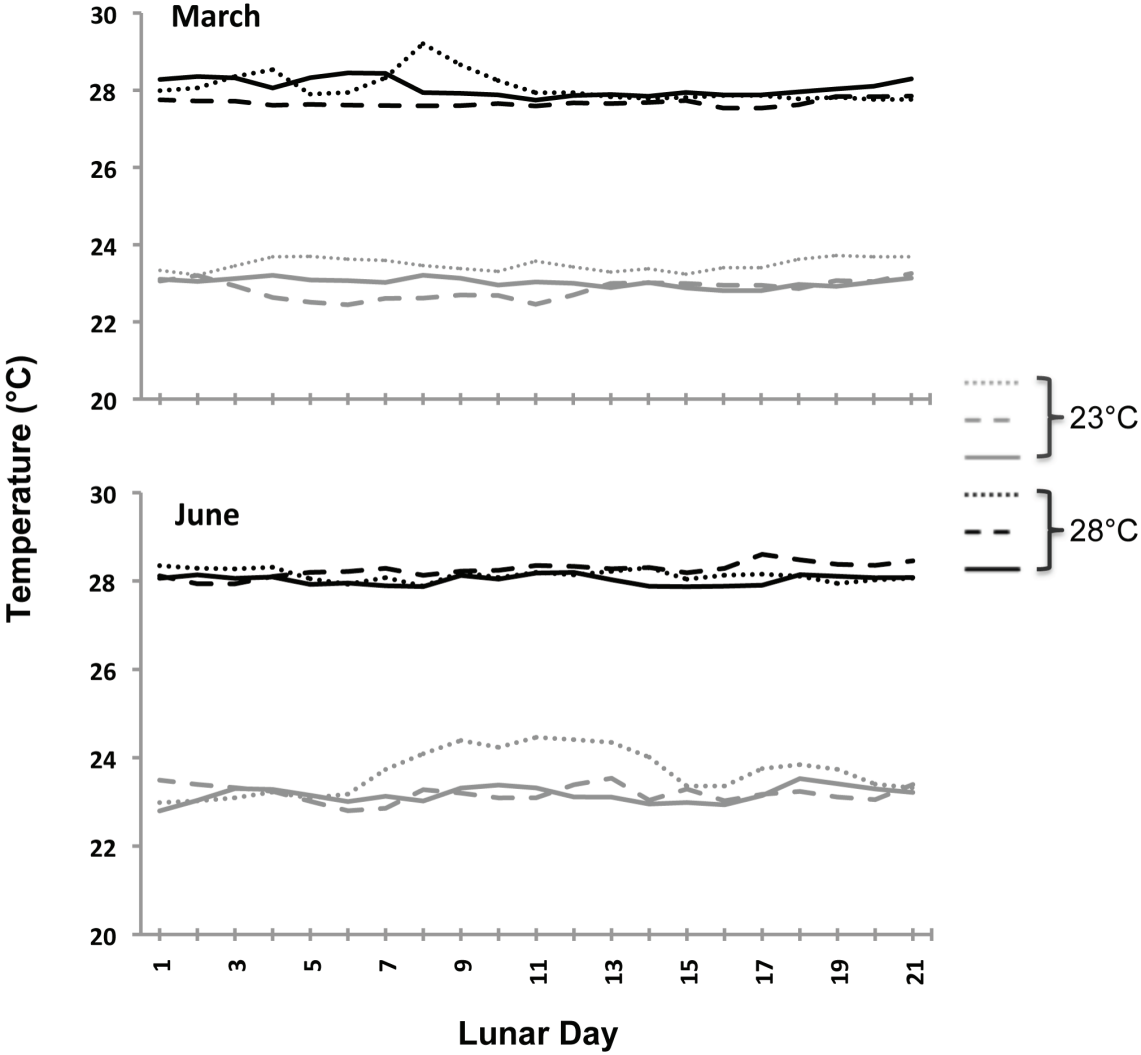
Average daily tank temperatures for both 23 and 28°C temperature treatments in lunar March and June 2012. Three lines represent temperature values in the three separate treatment tanks. Measurements were taken every 10 minutes and averaged for each lunar day.

Monitoring of planulation began on lunar day 1 (new moon), March 22, 2012 (lunar March) and July 19, 2012 (lunar June). Every evening during the lunar cycle, corals from each tank were individually placed into flow-through containers surrounded by 100 µm mesh plankton netting. Flotation devices were placed inside the netting to prevent nets from touching coral colonies. Each morning, nets were removed and the total number of planulae inside the netting was counted. Tank temperatures were recorded every 10 minutes using a HOBO temperature logger throughout lunar March and June 2012 (Figure 1).

### Data Analysis

The percent of the total number of planulae released each day was calculated as the total number of planulae released per individual colony per day (mean ± standard deviation, n = 6 colonies per temperature treatment) divided by the total number of planulae released by each colony during the monthly reproductive cycle. Raleigh’s test [29] was used to test the null hypothesis that planula release occurred uniformly throughout the lunar cycle. If the null hypothesis was rejected, then the mean lunar day (MLD) and angular deviation of planulation were calculated using circular statistics [19], [27], [29] to determine lunar timing of planulation. Circular statistic calculations were completed directly in an excel spreadsheet based on the equations and methods described in Zar et al. (1999).

A 1-way ANOVA was performed to compare MLD and angular deviation (AD) for each tank (n = 3), with average MLD and AD calculated for the two corals within each tank, within temperature treatments. A general linear mixed model with repeated measures was completed using a Poisson distribution with colony size treated as a covariate. Statistical analyses were completed using the statistical package R (R Foundation for Statistical Computing Vienna, Austria).

## Results

**Figure 2:**
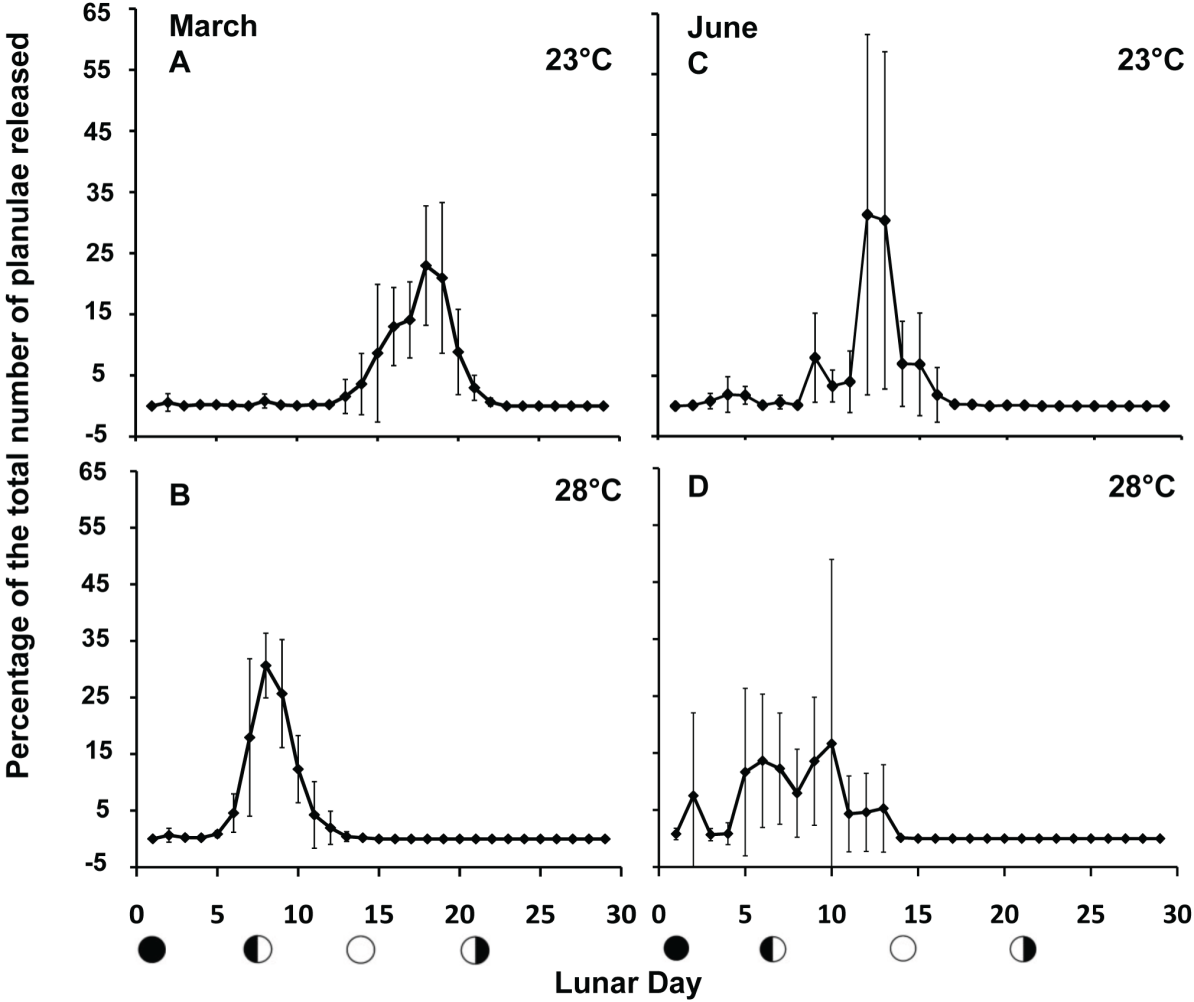
Percent of *P. damicornis* planulae released each day during lunar March (A and B) and Lunar June (C and D) 2012 reproductive cycles for colonies incubated at 23 (A and C) and 28°C (B and D). Points represent means of six colonies ± SE. Moon symbols represent lunar phases (new, 1^st^ quarter, full, and last quarter).

Raleigh’s test for uniform distribution indicated that planula release did not occur uniformly throughout the lunar cycle for either temperature treatment over both lunar March and June (p<0.001 for all tests) (Table 1). Differences were observed in the timing of planulation, measured as the percentage of planulae released per day with corals at 28°C releasing planulae earlier in the lunar cycle than those at 23°C for both lunar March and June (Figure 2). In lunar March, peak percentage of release occurred on lunar day 8 for the 28°C treatment, compared to lunar day 19 for the 23°C treatment. In lunar June, peak percentage of release was observed as two smaller peaks on lunar day 6 and 10 for the 28°C treatment, compared to a more significant peak at lunar day 12 for the 23°C treatment.

**Table 1:** Results of Raleigh’s test for uniform distribution of planula release by *P. damicornis* colonies during March and June lunar cycles.

	Lunar March		Lunar June	
	23°C	28°C	23°C	28°C
**MLD**	17.5±1.1	8.4±0.7	12.5±1.0	7.74±1.7
**Angular Deviation**	23.7±5.4	16.1±1.9	23.8±8.6	26.5±10.4
**z**	386–7261	2133–4638	93–208	11–291
**p**	<0.001	<0.001	<0.001	<0.001
**Planulae**	1,454±890	3593±1359	186.3±55	127±93
**Colony Size (cm)**	10.4±1.3	11.1±1.8	9.7±1.1	9.8±1.4

Mean lunar day (MLD) ± standard deviation, angular deviation of release ± standard deviation, range of Raleigh’s test statistic (z), p-value for Raleigh’s test (p), mean number of planulae released per tank ± standard deviation, and average colony diameter (cm) ± standard deviation.

MLD of planulation closely resembled patterns in percentage of planulae released whereas angular deviation, a measure of dispersion, was not significant in lunar March or lunar June, where there was a larger spread in days of release (Table 1). In lunar March, the average MLD and angular deviation of release were 17.5±1.1 and 23.7±5.4 for the 23°C treatment and 8.4±0.7 and 16.1±1.9 for the 28°C treatment, respectively. In lunar June, the average MLD and angular deviation of release were 12.5±1.0 and 23.8±8.6 for the 23°C treatment and 7.7±1.7 and 26.5±10.4 for the 28°C treatment, respectively. One-way nested ANOVAs showed significant differences in MLD (p<0.001) between temperature treatments in lunar March and significant differences in MLD (p = 0.014), between temperature treatments in lunar June (Table 2).

**Table 2:** Results from a 1-way ANOVA reporting the mean lunar day and angular deviation of planula release for lunar March and June.

			Lunar March					Lunar June		
Mean Lunar Day	DF	SS	MS	F	P	DF	SS	MS	F	P
Temperature	1	125.68	125.68	175.80	1.90 E-04	1	29.22	29.22	17.66	1.40E-02
Residuals	4	2.86	0.71			4	6.62	1.65		
**Angular Deviation**										
Temperature	1	85.43	85.43	5.93	7.2 E-02	1	11.00	10.96	0.107	0.76
Residuals	4	57.62	14.41			4	409.20	102.29		

Additionally, our results indicate that there were substantially more planulae released in lunar March (1,454±890 at 23°C and 3,593±1,359 at 28°C) compared to lunar June (186.3±55 at 23°C and 127±93 at 28°C) (Table 1). Significant differences in the total number of planulae released per lunar day were found to be associated with lunar day in both lunar March (p<0.001) and June (p<0.001) and colony size was not found to have a significant effect within individual lunar months (Table 3). Additionally, significant differences in the total number of planulae released per day were associated with temperature for lunar March (p = 0.001) but not lunar June (Table 3).

**Table 3:** Results from a general linear mixed model with repeated measures using a Poisson distribution with colony size treated as a covariate for the total number of planulae released per lunar day.

		Lunar March				Lunar June		
	Estimate	SE	Z	P	Estimate	SE	Z	P
**Intercept**	2.866	2.735	1.050	0.295	5.762	3.576	1.611	0.107
**Lunar Day**	−0.062	0.001	−61.030	2.00E-16	−0.082	0.004	−19.050	2.00E-16
**Temperature**	0.256	0.078	3.250	0.001	−0.116	0.093	−1.251	0.211
**Colony Size**	−0.400	0.292	−1.370	0.171	−0.026	0.262	−0.101	0.919

## Discussion

### Influence of Seasonality

In this study we observed a substantial difference in the total number of planulae released between lunar March and June 2012. This observed difference in the total number of planule released is likely due to seasonality. This hypothesis is consistent with previous findings showing that there were differences in planulae abundance between seasons in Pocilloporid corals in the Northwestern Philippines with dry seasons (March–May) having higher numbers of planulae released then wet seasons in (June–October) [20]. Differences in total number of planulae released in our study could also be attributed to colony size, since corals used in lunar March were, on average, 1 cm larger than those used in lunar June (Table 1). However, differences in planulation abundances are not expected to affect our results because timing of planulation was examined individually for each month.

### Timing of Planulation

Lunar periodicity has been correlated with the timing of reproduction for multiple coral species [2], [18] especially *P. damicornis*, where the timing of planulation is consistently linked to lunar phases [19], [20], [26], [27]. While lunar periodicity likely does play a role in the timing of reproduction, in some cases with good predictability, other environmental factors may be able to disrupt these cycles. In this study we directly tested the influence of low (23°C) and high (28°C) temperatures on reproductive timing, and show that changes in temperature have the capacity to significantly alter the timing of planulation. Clear differences were observed in the timing of planulation, shown as the percentage of planulae released each day (Figure 2), and temperature was found to have a significant impact on the MLD of planulation for both lunar March and June (Table 1 & 2). The observed variations in lunar day of release between temperature treatments suggests that elevated temperature modifies the affect of other cues, such as lunar periodicity, to drive timing of release.

Early release in the high temperature treatment suggests that temperature affects the reproductive physiology of the adult coral and/or the developing planulae, resulting in an acceleration of release [30]. This hypothesis is supported by a previous study on a broadcast spawning coral, *Echniopora lamellosa*, in Taiwan showing that reproductive processes, such as gametogenesis and spawning are plastic and can be accelerated by increasing seawater temperature [31]. Another study on Caribbean corals, within the genus *Madracis*, found that maturation of gametes was positively correlated with increases in seawater temperature, indicating that observed changes in timing could be attributed to internal cues associated with gametogenesis [32].

Alternatively, shifts in the timing of planulation could be a result of negative changes in planula physiology. This hypothesis is supported by multiple studies that have observed decreases in larval survival with elevated temperature. Larvae of the Hawaiian coral *Fungia scutaria* exposed to 27, 29, and 31°C showed gradual decreases in survivorship with animals incubated at 31°C having the highest rates of mortality [24]. Another study conducted on *P. damicornis* larvae in Taiwan found 28°C to be the thermal threshold for maximum respiration in planulae, with higher temperatures leading to reduced respiration and likely metabolic depression [33]. Additionally, a 5-fold decrease in survival was observed with a 1.5°C increase in temperature in *Acropora palmata* embryos and larvae from the Caribbean [11]. Although success of planulae after release was not assessed in this study, it has been shown that early-released planulae have lower settlement rates than those released later within a single reproductive cycle [19].

### Reproductive Plasticity

Our results reveal that temperature can act as a driver for plasticity in reproductive timing. Reproductive plasticity can enhance individual success in harsh or fluctuating environments [28]. While our study did not examine reproductive plasticity specifically in an adaptive context, we postulate that the phenotypic plasticity observed in this study may suggest capacity for an adaptive response to elevated temperatures in corals. This ability to shift reproductive timing in high temperature environments may indicate that climate change induced increases in ocean temperature may not be detrimental to reproduction, but rather simply alters its timing. Our findings also indicate that such shifts in timing can occur relatively quickly, as reproductive plasticity was observed over a single reproductive cycle. This is similar to findings observed in the coral *Echinopora lamellosa*, that show early spawning, when transplanted from colder northern to warmer southern Taiwan [19]. Understanding reproductive plasticity in corals is important because plasticity may provide corals the flexibility they need to be successful in a changing climate. However, many questions remain about how reproductive plasticity will influence the fate of corals, and their ecosystems, in the long term.

## Conclusions

Our results provide empirical evidence that a 5°C increase in temperature, accelerates the timing of planula release in *P. damicornis* in Nanwan Bay, Taiwan.

It important to note that the corals sampled for this study are of unknown genetic origin, and therefore could be clones of the same genotype. If so, this could decrease the variability in temperature response. Nonetheless, our findings reveal that there is plasticity in the timing of reproduction and these changes can occur rapidly, within a single lunar reproductive cycle. These results highlight the reality that increases in ocean temperature have the capacity to disrupt patterns of planulation in corals. Depending on how shifts in the timing of planulation correlate with other environmental variables and conditions, these alterations could lead to increased planulae mortality and decreased recruitment success, or may be indicative of the potential for adaptation to warming ocean temperatures. Understanding the affect of temperature on reproductive timing in *P. damicornis* provides information that can be used to predict patterns in reproductive success and colonization in a future of rapidly changing ocean climate conditions.
